# Modeling the Potential Spread of the Recently Identified Non-Native Panther Grouper (*Chromileptes altivelis)* in the Atlantic Using a Cellular Automaton Approach

**DOI:** 10.1371/journal.pone.0073023

**Published:** 2013-08-29

**Authors:** Matthew W. Johnston, Sam J. Purkis

**Affiliations:** 1 National Coral Reef Institute, Nova Southeastern University, Dania Beach, Florida, United States of America; 2 National Coral Reef Institute, Nova Southeastern University, Dania Beach, Florida, United States of America; University of Catania, Italy

## Abstract

The Indo-pacific panther grouper *(Chromileptes altiveli)* is a predatory fish species and popular imported aquarium fish in the United States which has been recently documented residing in western Atlantic waters. To date, the most successful marine invasive species in the Atlantic is the lionfish (*Pterois volitans/miles*), which, as for the panther grouper, is assumed to have been introduced to the wild through aquarium releases. However, unlike lionfish, the panther grouper is not yet thought to have an established breeding population in the Atlantic. Using a proven modeling technique developed to track the lionfish invasion, presented is the first known estimation of the potential spread of panther grouper in the Atlantic. The employed cellular automaton-based computer model examines the life history of the subject species including fecundity, mortality, and reproductive potential and combines this with habitat preferences and physical oceanic parameters to forecast the distribution and periodicity of spread of this potential new invasive species. Simulations were examined for origination points within one degree of capture locations of panther grouper from the United States Geological Survey Nonindigenous Aquatic Species Database to eliminate introduction location bias, and two detailed case studies were scrutinized. The model indicates three primary locations where settlement is likely given the inputs and limits of the model; Jupiter Florida/Vero Beach, the Cape Hatteras Tropical Limit/Myrtle Beach South Carolina, and Florida Keys/Ten Thousand Islands locations. Of these locations, Jupiter Florida/Vero Beach has the highest settlement rate in the model and is indicated as the area in which the panther grouper is most likely to become established. This insight is valuable if attempts are to be made to halt this potential marine invasive species.

## Introduction

### 1.1. Invasive species in the Atlantic

Marine invasive species are much less common than their freshwater counterparts; however, sightings of non-native species in Atlantic waters have been well documented by the United States Geological Survey Nonindigenous Species (USGS NAS) database [1] and most are thought to be isolated aquarium releases [2]. One species is the Indo-Pacific lionfish (*Pterois volitans/miles*), a very successful invader now established throughout the Caribbean, Gulf of Mexico, and Atlantic coasts as far north as Cape Hatteras, NC, USA [3]. A thorough analysis of the lionfish invasion, based on records from the USGS NAS database, was presented by [4] and an algorithm developed (the Invasionsoft Model – ISM) which is useful for predicting the spread of invasive species, like the lionfish, which exhibit fidelity to ranges in sea temperature, salinity, and water depth.

**Figure 1:**
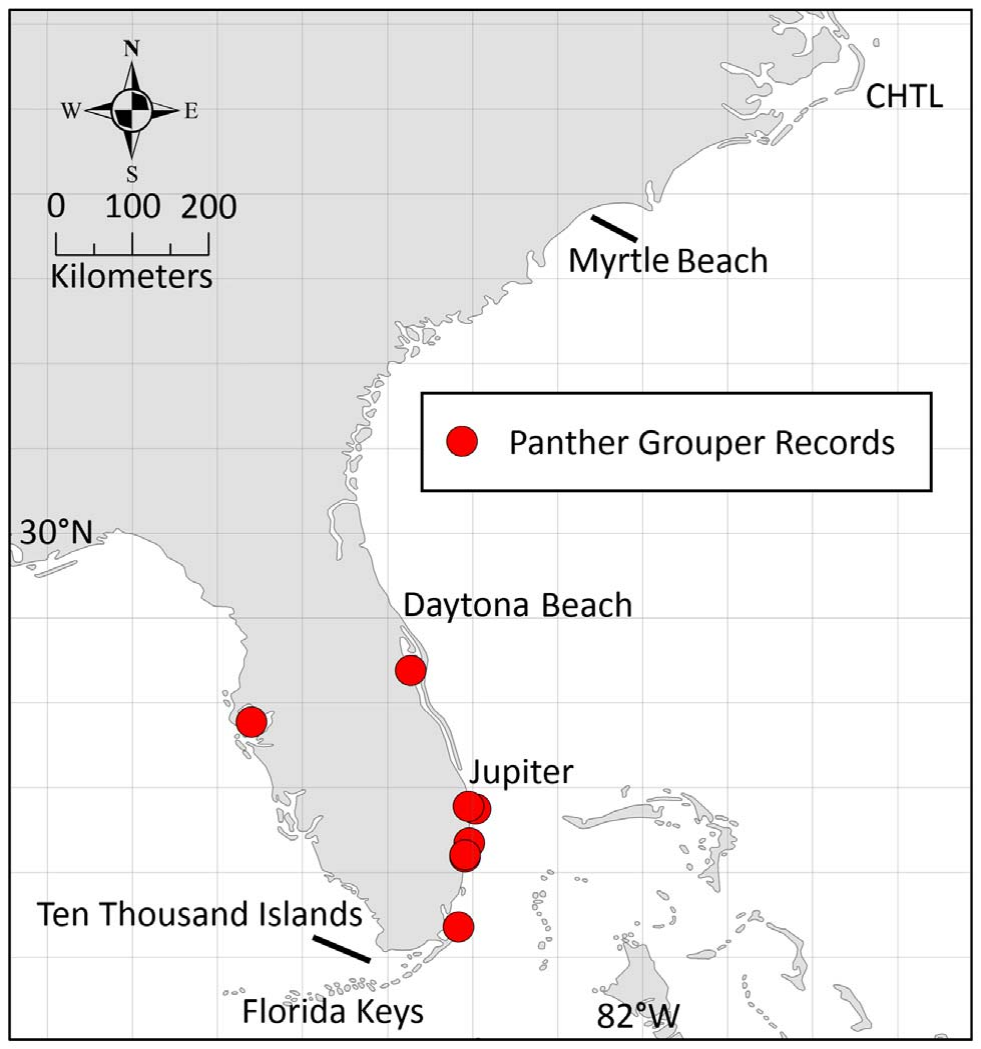
Panther grouper records. Records from the USGS NAS indicating locations of panther grouper captures or sightings.

The panther grouper (*Chromileptes altiveli*), sometimes termed the “humpback grouper” or “barramundi cod”, is an exotic and potentially invasive species that has been documented seven times in the Atlantic, with one report from the Gulf of Mexico, since 1994 [1] (Figure 1). Six of the seven records from the Atlantic were recorded in the last ten years, indicating sightings of this species are becoming increasingly common and suggesting that this Indo-pacific tropical species has the potential to follow in the footsteps of the lionfish and become the next large-scale invader of Atlantic waters.

### 1.2. Panther Grouper Species Profile

The panther grouper is an Indo-pacific predatory fish species found in lagoons, hard bottom habitats, and seaward well-developed coral reefs, in depths up to 40 m [5]. The panther grouper attains a size of approximately 70 cm, a weight of 7.0 kg, and lives up to 19 years with a potential reproductive life of 17 years (females are reproductively viable at a weight of around 1 kg, 15.5 cm, and 18 months) [6]. The panther grouper is a popular aquarium fish due to its white with black polka-dot coloration as a juvenile and occupies a trophic level similar to native Atlantic snapper and groupers (consuming small teleosts and crustaceans) [5].

### 1.3. Panther Grouper in Comparison to Lionfish

The panther grouper shows many potential invasive characteristics and shares ecomorphology and a breeding strategy similar to the efficacious Atlantic invasive species, the lionfish. The panther grouper and lionfish are also both Indo-pacific apex reef predators [7], [5], [8]. Two of the USGS NAS panther grouper records indicate sightings of the same individuals in the same location over a period of weeks or months, implying site fidelity – another trait in common with lionfish [9], [10], [1]. Neither lionfish nor the panther grouper have been studied in detail in their native range as they are relatively benign species. In contrast to lionfish, the panther grouper is a protogynous hermaphrodite [6]. In monosex situations, such as may occur with an introduced population containing few individuals, the female may transition to a male [11]. The panther grouper breeds year round in captivity on a monthly cycle before and after the new moon, with a peak in natural spawning between October and January [12], [6]. Eggs are buoyant and are broadcast, relying on currents for advection similar to the lionfish. In a captive study, quantity of eggs produced ranged between.2 to 1.2×10^3^ and fertilized eggs hatched in about 18–20 hours at a temperature of 28–29°C [11]. Larval duration of the panther grouper is estimated to be around 40 days, which is consistent with the range of larval duration values for marine fish estimated by [13] and the same as the similar native Atlantic soapfish grouper *Rypticus saponaceus* [14]. Percentage survival of larvae until the age of 50 days was highly variable, from 2.63% to 53.90%, in highly controlled artificial conditions [15]. Larval mortality rates for wild panther grouper populations have not been documented. Table 1 compares the reproduction, vagility, and life strategies of both species.

**Table 1:** Panther Grouper verses Lionfish.

Life-History Characteristic	Panther Grouper	Lionfish
*Trophic level*	apex predator – teleosts, crustaceans	apex predator – teleosts, crustaceans
*Adult size*	70 cm, 7.0 kg	10 cm, 300–400 g [25]
*Longevity*	19 years	up to 30 years in captivity [26]
*Defenses*	coloration	coloration, venomous
*Site fidelity*	likely moderate	high
*Thermal tolerance*	16°C	10°C
*Breeding strategy*	protogynous hermaprodite	monogametic
*Reproductive age*	18 months	12 months
*Egg type*	floating, broadcast	floating, contained in a mucous sac
*Larvae type*	pelagic	pelagic
*Quantity of eggs*	0.2 to 1.2 million	>2 million annually [27]
*Breeding season*	year round with a peak October – January	year round [27]
*Breeding cycles per month*	up to 4	up to 7.5 [27]
*Larval duration*	40 days	20 to 35 days [28]

Comparison of life history and reproductive traits of panther grouper and lionfish.

A significant dissimilarity between lionfish and the panther grouper are the morphological differences that lionfish exhibit from native Atlantic teleosts. Lionfish morphology is completely unique with expansive, venomous striped pectoral and dorsal fin rays – unlike any extant species in the Atlantic [7]. Contrariwise, panther grouper share a body form and function similar to other native Atlantic grouper species like the soapfish (*Rypticus saponaceus*) and marbled grouper (*Dermatolepis inermis*). As such, the postulation is made that this unique lionfish morphology lends a positive advantage in both predation and predator avoidance, potentially negating any morphological-based advantage in favor of the panther grouper.

### 1.4. Purpose

This paper presents a suite of simulated scenarios that describe the potential spread of the panther grouper in the Atlantic, should a breeding population become established, based on the ISM previously utilized studying lionfish [16]. Using the proven modeling technique, this study is the first known prediction of the potential spread of panther grouper in the Atlantic, presented at a critical time before the establishment of a breeding population. The cellular-automaton model examines life history characteristics of the species, including fecundity, mortality, and reproductive potential, combined with physical oceanic parameters, to describe the spread of this potential new invasive species. The findings in this study are presented as a first indication of the possible settling areas of breeding populations, given ideal conditions, with the intent that this may be used as a guideline for monitoring and first-response efforts. As such, simulations were analyzed for 1,000 random locations within 1° of USGS NAS capture records of panther grouper to identify potential “hot spots” of future establishment of the species. Should one or more breeding populations become established in the study area, our work can be used to guide a coordinated response to a panther grouper invasion, as opposed to the *ad hoc* approach used for lionfish control. Additionally, two case study locations were examined in detail; the Florida Keys, Florida, USA (CS_FK_), and in Broward County, Florida, USA (CS_BC_). Herein is presented a potential proposed timeline of the future spread of the panther grouper through the Atlantic, including predictions for the sequence of invaded localities.

## Methods

### 2.1. Processing Logic and Model Inputs

Cellular automata (CA) models, such as the ISM, consist of four elements; *conceptual cells*, *cell state*, *neighborhood cells* and a set of *rules*. In a CA model, the study area is divided into a lattice of spatially explicit *conceptual cells,* each of which contains unique parameter values. One founder cell is initially marked settled (the *cell state*) and subsequent cells in the *neighborhood* are marked settled based on an acceptable range of values including a stochastic variable (the *rules*). In the ISM, a proportional weight factor (part of the CA *rule*) is assigned to each parameter and is used to determine influence on that cell (the CA *conceptual cell,* in the CA *neighborhood*) meeting the conditions for settlement (the CA *cell state*). The CA algorithm is repeated for each settled cell for a pre-determined number of cycles, with the result being a list of latitude/longitude points and the cycle in which settlement occurred. A complete in-depth discussion on the step-by-step mechanics and technology used of the base ISM are discussed in [4].

The initial version of the ISM excluded the temporal aspect of an invasion, instead focusing on the chronology of spatial occurrences. To include periodicity in the ISM, the model now integrates the timing of species life-history components, which are critical to predicting the progression of an invasion [17]. Ocean current, depth, and sea surface temperature have been retained and chlorophyll concentration added as baseline data inputs. In addition, more granular physical parameter data have been compiled, enabling the model to perform simulations to a scale of approximately 4 km in the center of the study area. Also included are the temporal parameters of larval duration, breeding age, and mortality to present a time-scale of the likely spread mechanics of an invasion. New to the ISM is the use of kernels, which are representative units taking the place of finite quantities of individual propagules. These kernels are acted upon independently in each cycle and undergo advection and diffusion, imparting separate movement more illustrative than the previous ISM. This is similar to methods used by [18], substituting lagrangian movement with a cellular automaton approach. Following is an examination of parameters used in the ISM and their initial data sources (Table 2).

**Table 2:** ISM parameter inputs.

Parameter Name	Value	Rationale	Source
*Cycles (months)*	60		
*Grid Size*	6 Arc Minutes	10 fold increase in granularity from previous lionfish study	[4]
*Sea Surface Temperature Range*	16° C – 32.820°C	based on temperature extremes in their documented native range	[29]
*Sea Surface Temperature Weight*	.02	parameter does not largely influence initial distribution for a current-dispersed species	[4]
*Chlorophyll Range*	.10 – 99.981 μgL^−1^	chlorophyll concentrations on two sections of the Great Barrier Reef, a native habitat for PG, indicated a mean concentration of 0.2μgL^−1^ and 0.54 μgL^−1^ – lower limit of 0.10 μgL^−1^ based on comparative concentrations in its native Australia and similar concentrations in the Atlantic	[30]
*Chlorophyll Weight*	.02	parameter does not largely influence initial distribution for a current-dispersed species	[4]
*Depth Range*	1–40 M	known to inhabit lagoon type areas and shallow reefs to a depth of 40 meters; parameter does not largely influence initial distribution for a current-dispersed species	[29]
*Depth Weight*	.02	parameter does not largely influence initial distribution for a current-dispersed species	
*Current Weight*	.90	the most influential parameter to the spread of similar invasive lionfish	[4]
*Propagule Duration*	40 days	durations documented by [13] and that of an ecomorpholigically similar native Atlantic soapfish	[14]
*Propagule Mortality (Z_p_)*	0.2 d ^−1^	In marine teleosts, larval *Z_p_* varies widely from 0.01 d^−1^ to 0.69 d^−1^ as reported by [13]. As a default baseline for the ISM, a *Z_p_* rate of 0.20 d^−1^ is used based on connectivity studies reported by [18], which are derived from [13]. Given the variability of larval mortality rates reported in captive populations [11], and unknown wild mortality rates, the rate chosen is a reasonable proxy. This same rate was used by [18] to model connectivity patterns, based on a tropical damsel species with pelagic larvae for the Caribbean region.	[18], [13],[11]
*Breeding Age*	18 months	documented in cultured conditions at approximately 18 months and 15.5 cm length	[6]
*Mortality (Z)*	.26 y ^−1^	based on two locations in Australia, the Great Barrier Reef and Torres Strait.	[6]
*Propagule Quantity*	15,000	fertilization rates are estimated at 0–90% and hatching rates usually exceed 30% – estimated viable propagules per cycle (25%×200,000 (fertilization rate) ×30% (hatching rate)) based on natural reproduction, as opposed to controlled breeding situations in ideal circumstances	[31], [15]
*Kernel Count*	20	20 – resulting in a larvae/kernel ratio of 0.0013 (approximately 750 larvae per kernel)	
*Breeding Cycle Begin/End*	January/December	natural reproduction has been documented year round	[6]
*Monthly Breeding Cycle*	30 days	breeding occurs on a monthly cycle around the full moon; conservatively, value has been set to one breeding session monthly	[12]
*Starting Month*	January	arbitrary starting month	

Input values for all parameters considered in the ISM, including their source.

#### 2.1.1. Static Parameters (Ocean Current, Sea Surface Temperature, Chlorophyll Concentration, Ocean Depth)

As documented in [16], the ISM uses a weighted value system to determine the influence of static parameters on the temporal spread and eventual setting of propagules. These weight factors are proportional to one other, and are standardized to a value between zero and one in the ISM algorithm.

The initial version of the ISM (using the default parameter set) examines a geographic area encompassing the western Atlantic Ocean, Caribbean Sea, and Gulf of Mexico from 45° to 5° N latitude and −100° to −50° W longitude, which corresponds to the approximate geographic extent of the lionfish invasion. In the enhanced ISM, the eastern Pacific is included for an area encompassing 50° N to 40° S latitude and −140° to −20° W longitude. For the purposes of this study, the panther grouper is presumed contained for the simulation duration to the Atlantic Ocean, Gulf of Mexico, and Caribbean.

- *Ocean Current (OC):* The OC data used in the ISM are based on values from the HYCOM ocean model [19]. HYCOM is a compilation and forecast of global ocean currents based on in-situ measurements and remotely sensed data. The measurements used in the model are granular to 1/12° which is roughly 8 km at the center of the study. A representative year (2005) was chosen as the basis for the model and monthly mean velocity and current angle were compiled based on daily projected values.
- *Sea Surface Temperature (SST):* Compiled SST estimates are based on MODIS data. These remote sensing data were compiled to a level 4 km in the center of the study area on a monthly mean basis for the representative year 2005.
- *Chlorophyll Concentration (CC):* Compiled CC values are based on MODIS data and are a proxy for primary productivity. Data were compiled to a level 4 km in the center of the study area, on a monthly mean basis for the representative year 2005.
- *Ocean Depth (OD):* OD data are sourced from the ETOPO1 1 Arc-Minute Global Relief Model which combines bathymetry and topography data based on underway hydrographic soundings and satellite altimetry estimates [20]. Data were compiled to a level of 4 km in the center of the study area.

#### 2.1.2. Fecundity Parameters

The quantity and quality of eggs and larvae released are critical components when determining fecundity of a species [17]. The following factors are considered when running the ISM, all of which contribute to the fecundity of a species and serve to impart a time scale.

- *Propagule Duration:* The approximate duration of larvae, from the initial spawning to the eventual settling point.
- *Propagule Mortality (Z_p_):* The larval mortality rate for the propagule duration period.
- *Breeding Age:* The minimum age (in months) at which an established adult in the ISM is eligible to contribute propagules to the model.
- *Mortality (Z):* The adult mortality rate which is applied to established populations after the propagule duration period.
- *Propagule Quantity:* The quantity of propagules per breeding cycle per individual, defined in this study as viable larvae.
- *Kernel Count:* The number of kernels, representing multiple tangible propagules.
- *Breeding Cycle Begin/End* ***:*** The beginning and end of the breeding cycle, signifying which months breeding is likely to occur.
- *Monthly Breeding Cycle:* The number of times per month the study species breeds.
- *Starting Month (SM):* The start month of the simulation.

**Figure 2:**
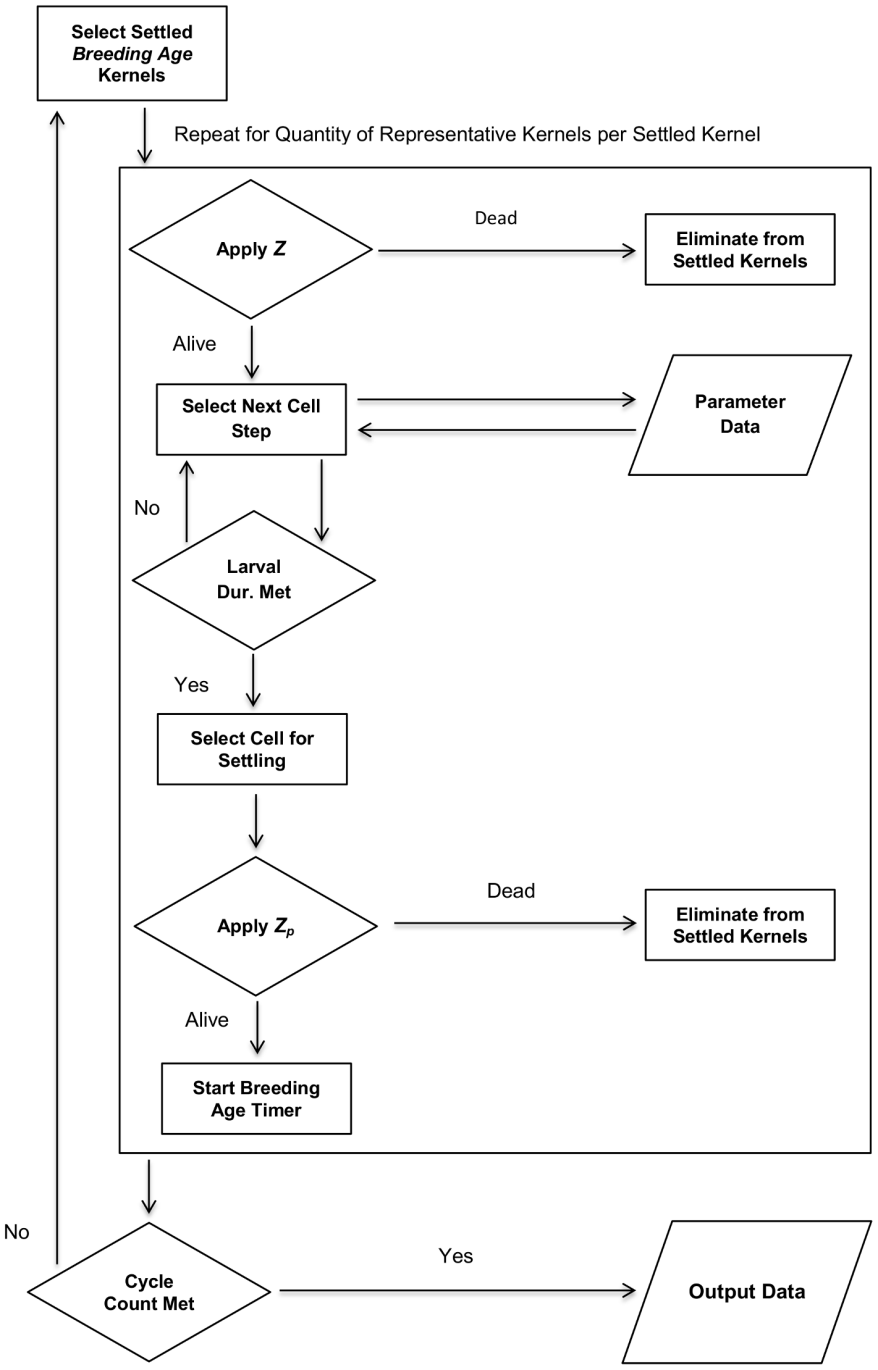
Process flow of the enhanced ISM.

Figure 2 presents an overview of the algorithmic flow in the model. Simulations have a definitive start and end time, expressed as a starting month and run cycles for a period of months. The ISM tracks the applicable month when selecting OC, SST, and CC values from the database. From the initial location, individual kernels are acted upon to determine the next likely geographic step, based on the grid lattice being used, and physical parameters values present in the cell. Ocean current velocity values are largely determinate of the temporal spread to downstream grid cells, with temperature, depth, and chlorophyll having a lessor influence. In grid cells with low current velocity, the effect on cell score by other static parameters, like temperature and depth, is effectively increased. This is due to the proportional decrease in total cell score contribution by ocean current [16]. A running sum is calculated to track transition time and once the larval duration threshold is reached, the last cell is selected as a settling point for the kernel. The ISM then applies *Z_p_* to determine kernel survival during transport, and examines SST, OD, and CC to determine if the cell value falls within the designated inhabitable value range. If a cell is selected for settling, a breeding age cycle timer is started to designate when the settled kernel (representing a juvenile at this point) is eligible to contribute larvae to the model. From the pool of settled kernels for each cycle, a random number between zero and one is selected to determine *Z* of the kernel. If the random value falls below *Z*, the kernel is flagged ineligible to contribute (death). If the kernel has reached maturity, as defined by the breeding age, the kernel is flagged as a breeding kernel and begins contributing larvae on the next cycle. Breeding kernels are eligible to contribute larvae on each cycle until selected for elimination by the *Z* test.

### 2.2. Test Cases

To identify potential settling locations (“hot spots”) regardless of origination in southern Florida, a composite simulation was created by selecting 1,411 points representing all grid locations (at a scale of 6 arc minutes or roughly 10 km) within 1° of USGS NAS panther grouper records (excluding the Gulf of Mexico record) and a water depth limit of 40 m. From these locations, a random number generator was used to select 1,000 points. One simulation was then created for each position, eliminating bias as to the exact introduction point. Two detailed test case scenarios were also chosen for closer examination to demonstrate differences between a south Florida (CS_BC_) and Florida Keys (CS_FK_) breeding population. A simulation duration of 78 months was deemed sufficient to illustrate the initial spread pattern and provide settling location guidance for all simulations. Table 1 shows the input values used for each ISM parameter with the source of the data as noted.

### 2.3. Model Validation

In [4], an aggregate Receiver Operating Characteristic (ROC) analysis and resulting Area Under the Curve (AUC) value was calculated to account for false positive/false negative predicted sequences based on a best fit model. In order to perform this analysis, a historical invasion pattern must be present. The USGA NAS records for panther grouper likely indicate that the species has not yet established itself in the study area, though it has been documented over a number of years. Likewise, the study does not analyze a historical invasion sequence for this species (consequently a ROC and AUC cannot be calculated for this study) and relies on the USGS NAS records solely to delineate potential sources of initial breeding population locations. To test precision and demonstrate that the model is not purely random (the null hypothesis, H_o_), a probability distribution of spread was produced by creating 20 simulations with the same input parameters for the two detailed case studies, CS_FK_ and CS_BC_. H_o_, in this scenario, is defined as a simulation with purely random spread of kernels based singularly on a stochastic variable. H_o_ simulations for each case study were created by selecting the same origination points used in each study and running the model excluding the influence of current, chlorophyll, depth, temperature, and all fecundity parameters on the resulting spread. H_o_ simulations were run until all locations in the study area contained established populations, allowing the temporal sequence of each simulation to be analyzed. By eliminating all influencing variables, this presents a truly random spread pattern from the origination point. Following the *Caulerpa taxifolia* example in [16], the sequence of spread for each simulation was then recorded using grid quadrants at a 0.5°×0.5° scale. To analyze the overall pattern of invasion, the quadrants were summed across all simulations and counted for the first 12 invasion steps (defined as establishment of a breeding population in one grid quadrant) for CS_FK_ (H_FK_) and 10 steps for CS_BC_ (H_BC_). The number of steps reflects the count of occupied grid quadrants common to all simulations in each respective case study. The quadrant with the highest count for each step was selected as the representative cell for that step. Next, each individual simulation was compared to the overall representative sequence and summed based on adherence to each step. The simulation with the greatest sum was then selected as the Representative Model (RM). To evaluate any relationship between the detailed simulations and H_o_, a Spearman's Rank Correlation Coefficient (ρ), a standard metric to test correlation, was produced comparing the RM to H_o_ in the same manner as [16] where a ρ value of one indicates a perfectly monotonically related result and a value of zero shows no relationship. For an *n* of 12 for H_FK_, with a two-tailed 0.05 significance level, a critical value of 0.59 was selected based on *n* – 2 degrees of freedom (*df*), and for an *n* of 10 (H_BC_), a critical value of 0.65 was designated based on [21]. Finally, ρ values were calculated for all 20 simulations in each model run between the individual simulation and the appropriate RM to test correlation and significance, and a mean ρ value computed.

### 2.4. Sensitivity Analysis

Larvae survivorship in fish population models is inherently sensitive to small changes in the larval mortality rate, resulting in a pronounced effect on larval recruitment [13]. Because larval survivorship for this species has not been documented in the wild and mortality is likely one of the most variable and influential parameters in the study, both case studies were modeled varying *Z_p_*±10% (values of 0.18 d ^−1^ and 0.22 d ^−1^), with all other parameters equal, to test sensitivity to this parameter. Results from these 20 alternate simulations were analyzed in the same manner as the original studies_._ The RM chosen for each alternate scenario was then compared to the case study's original RM, using the SRCC method to evaluate correlation of the invasion sequence steps, resulting in a ρ value for each alternate scenario. This method conveys correlation of invasion sequences for the alternate RMs to the original RM when mortality rates are varied. Finally, settlement locations for each alternate scenario were summed and projected on a map, illustrating relative settlement concentrations and patterns.

## Results

### 3.1. Composite Simulation Case Study

**Figure 3:**
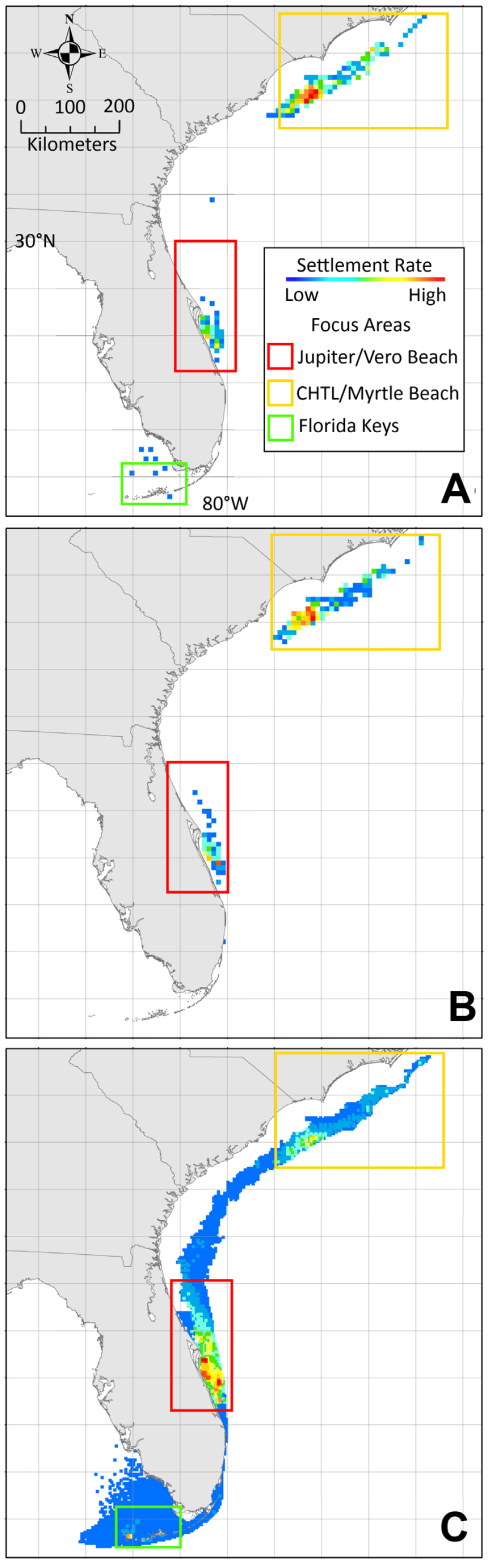
Settlement and focus area maps. Settlement rates of adult breeding populations for panther grouper on a ‘hot’ (red) to ‘cold’ (blue) scale using Jenks' natural breaks as class divisions (a method that reduces inter-class variance and maximizes variance between distinct classes) for CS_FK_ (A), CS_BC_ (B) and composite study (C) simulations for a duration of 78 months. Focus areas for early detection are indicated for the Jupiter Florida/Vero Beach (red), Cape Hatteras Tropical Limit/Myrtle Beach (orange), and Florida Keys/Ten Thousand Islands (green) locations.

Settling sites from the composite simulation study were summed per location and projected on a map (Figure 3). The composite simulation indicates three potential hot spots, presented in order of relative potential for establishment; 1) the neritic zone north and west of Jupiter Florida, centered near Vero Beach Florida (∼27.250° N to 29.500° N to a depth of 40 m), where the Gulf Stream diverts from the coastline and the continental shelf extends northward, 2) offshore South Carolina, centered near Myrtle Beach with a northern limit just south of the Cape Hatteras Tropical Limit (CHTL), as described in [4] (∼32.500° N to 34.850° N, −80.000° W to −75.700° W in depths <40 m), and 3) the lower Florida Keys extending into the Ten Thousand Islands area off the tip of south Florida (∼24.500° N to 25.000° N, −82.250° W to −81.500° W in depths <40 m). For the top twenty locations with the highest settlement rate by count (all near Vero Beach), the mean month of establishment was approximately 66 months which represented 17.5% of all kernel counts.

### 3.2. Case Study One – Florida Keys, South Florida

CS_FK_ assumes a breeding population of panther grouper in the Florida Keys. The USGS NAS records presently indicate a large specimen recently captured in the Florida Keys and the coordinates of 24.583° N and −81.217° W were chosen as an initial breeding population location. CS_FK_ agrees with the composite simulation regarding settling points of larvae in the initial stages of an invasion. In this scenario, most larvae are transported east and north on the Gulf Stream current, eventually settling in two primary locations; 1) just south of the CHTL, and 2) north and west of Jupiter Florida near Vero Beach (Figure 3). From the model, and based on HYCOM current data, weak meandering currents tend to concentrate larvae that have departed the Gulf Stream near this location. OD, SST, and CC values in both locations are well within tolerances for this species.

Due to the Florida Keys origination, CS_FK_ also indicates a potential spread into the Ten Thousand Islands area off the tip of south Florida, where all parameters are within range for settling to occur. By year four, breeding populations exist at the CHTL, Jupiter Florida/Vero Beach, and Florida Keys/Ten Thousand Islands locations according to the model.

### 3.3. Case Study Two – Broward County, South Florida

CS_BC_ assumes a breeding population of panther grouper off the coast of south Florida in Broward County. The coordinates of 26.217° N and −80.083° W were chosen as the point of establishment for the initial breeding population as the USGS NAS indicates several records near this location. CS_BC_ indicates that two of the same locations (south of the CHTL and Jupiter Florida/Vero Beach) have potential as settling points of larvae in the initial stages of an invasion (Figure 3). In this second scenario, most larvae are once again transported north on the Gulf Stream current. Past the maturation period of 18 months, settled juveniles occur near both the locations, and by year four, breeding populations are established south of the CHTL and near Jupiter Florida/Vero Beach. Spread into the Ten Thousand Islands area was not forecast by the model for this scenario.

**Figure 4:**
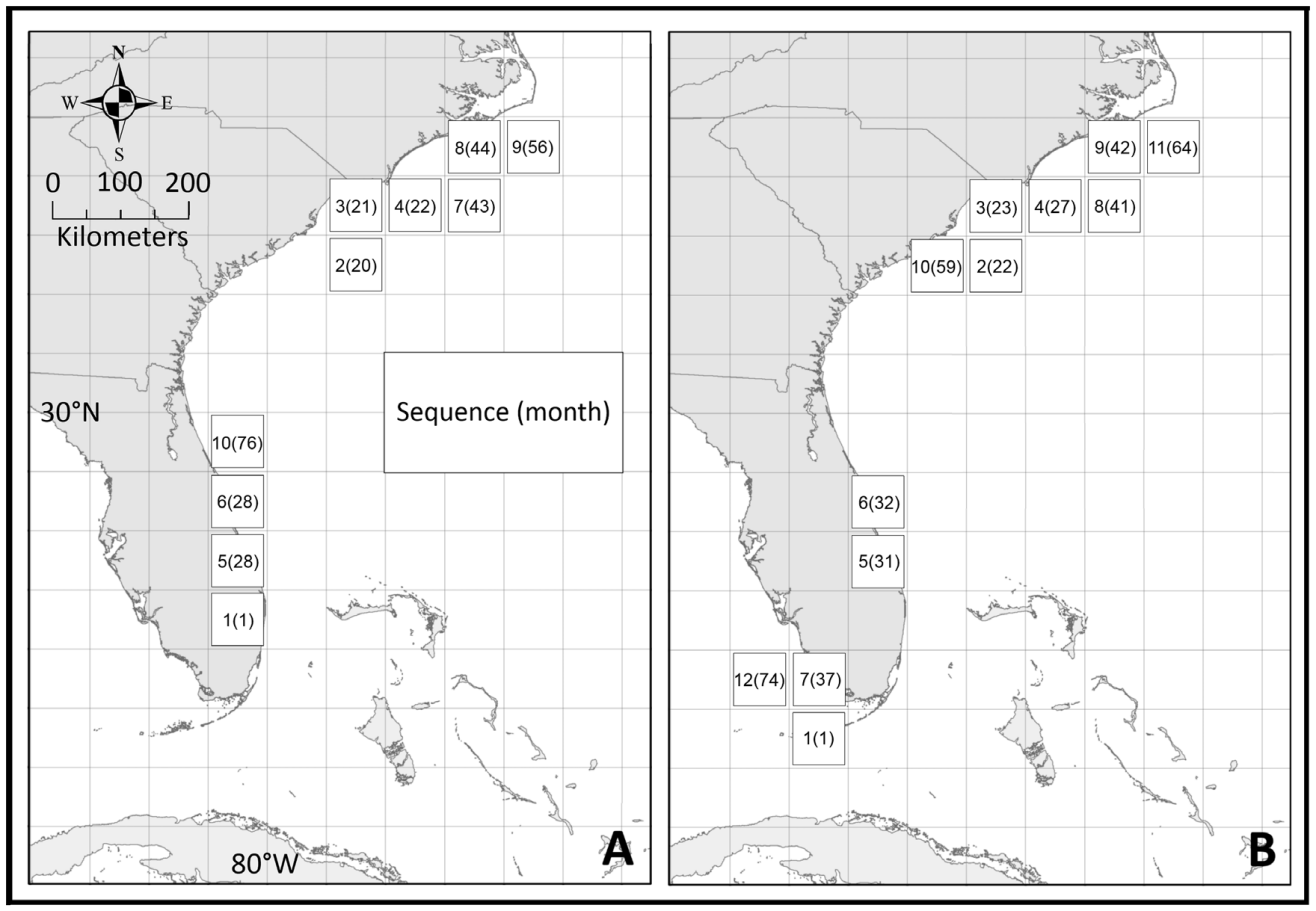
Temporal-spatial progression map. Map indicating the sequence and relative month [sequence(month)] for the first 10 steps for a Broward County origin (A) and a 12 steps for a Florida Keys origin (B).

The ISM indicated initial settling of larvae (non-breeding populations) 6–9 months after establishment of a breeding population in both CS_FK_ and CS_BC_. The model predicts breeding populations of panther grouper would develop first in the northernmost CHTL settling point (month 20–22), followed secondly by Jupiter Florida/Vero Beach (month 28–31), and lastly, for CS_FK,_ the Florida Keys/Ten Thousand Islands location (month 37) (Figure 4). In both cases, the northernmost limit for the panther grouper is likely just south of CHTL, as overwintering temperatures drop below the predicted 16°C thermal tolerance. This is slightly south of the projected potential distribution of lionfish, which have a documented tolerance to 10°C [22]. Also notable is the lack of settling in the near-shore neritic zone roughly north of Daytona Beach, Florida to the CHTL, where winter SSTs drop below panther grouper tolerances. Due to strong near shore currents from the Gulf Stream, limited settling occurred off the south Florida coast between the upper Florida Keys and Jupiter Florida (Figure 3).

It has been shown that coral reefs of the Florida Keys and south Florida show weaker connectivity to Bahamian reefs than would be expected based on distance alone, and are rather more closely associated with the upstream Mesoamerican Barrier Reef [23], [18]. Strong currents from the Gulf Stream act to transport larvae away from this area and also act as a barrier to conveyance across the stream to the Bahamas as shown in the ISM and transition matrixes by [18]. In the model lionfish case and as documented by USGS NAS records, initial lionfish records in south Florida were recorded at least 10 years before those in the Bahamas [4]. Accordingly, a crossover event did not occur in the timeframe examined for the initial input values.

### 3.4. Model Validation

**Figure 5:**
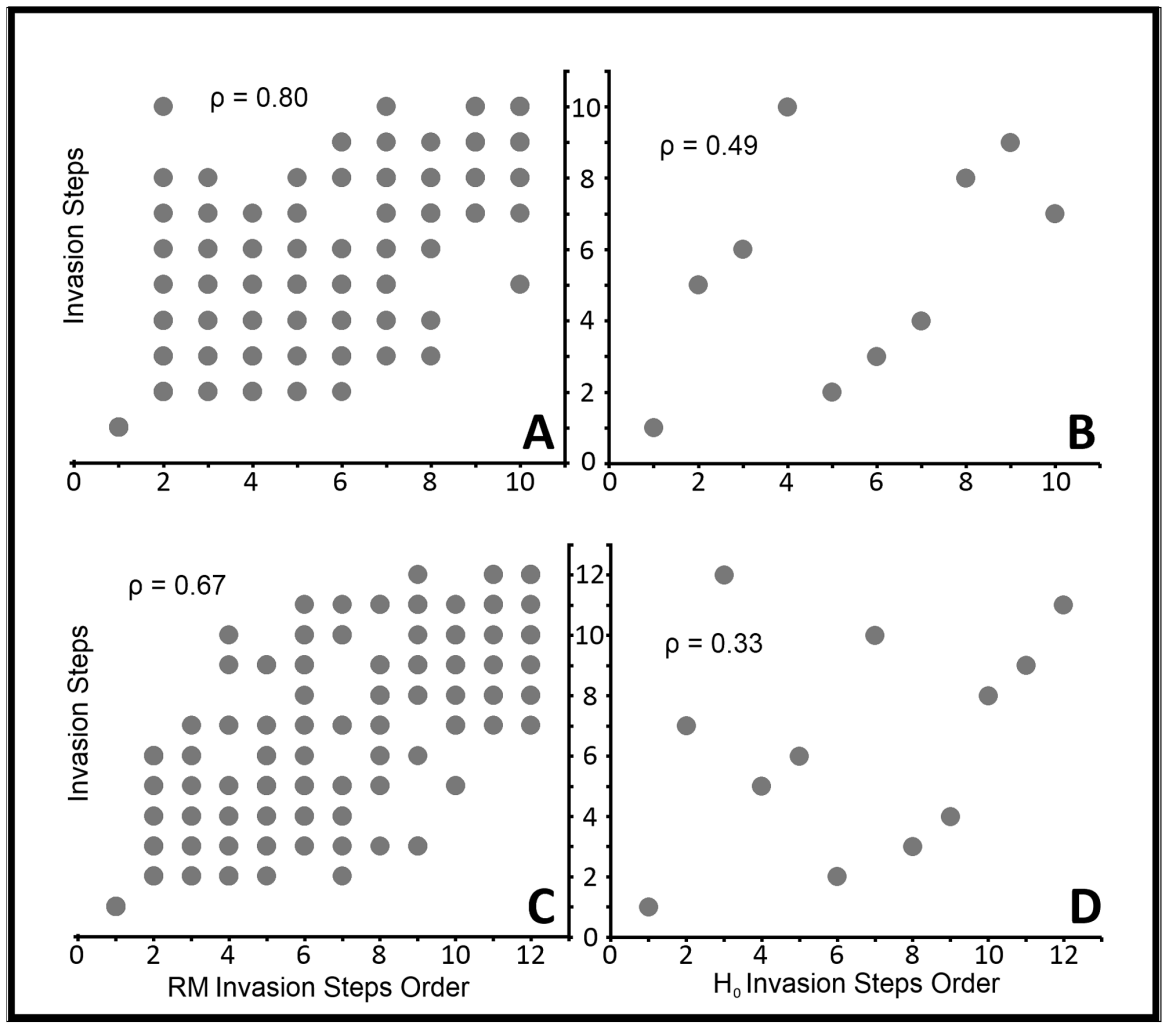
Spearman's Rank Correlation Coefficient (SRCC) calculations. SRCC calculation with a ρ = 0.80 for CS_BC_ when comparing 20 individual model runs (y-axis) to the RM (A), and ρ = 0.49 when comparing the RM (y-axis) to H_o_ (B). SRCC with a ρ = 0.67 for CS_FK_ when comparing 20 individual model runs (y-axis) to the RM (C), and ρ = 0.49 when comparing the RM (y-axis) to H_o_ (D). X-axis indicates the sequential order of establishment for the RM, and the y-axis indicates the order of establishment for each comparative simulation. Perfect correlation (SRCC of 1.0) is indicated by a point lying precisely on the diagonal from bottom-left to top-right.

To validate the ISM, 20 identical simulations were created for CS_FK_ and CS_BC_ using the parameter input values for this study (Figure 5). The ρ value calculated comparing H_o_ to each RM was 0.33 for H_FK_ and 0.49 for H_BC_. Using a significance level of 0.05 and resulting critical value of 0.59 (H_FK_) and 0.65 (H_BC_), correlation values for both models proved to be insignificant when compared to H_o._ When evaluating the mean of 20 simulation runs for each model compared to the representative RM, a ρ of 0.67 (H_FK_) and 0.80 (H_BC_) were calculated, respectively. From the results of the significance tests, the ISM shows no monotonical relationship to H_o_ and the mean of each model run is significantly correlated when comparing to the selected RM. These findings indicated that the ISM is not purely random and repeated simulations using the same inputs show highly similar and significant results.

### 3.5. Sensitivity Analysis

**Figure 6:**
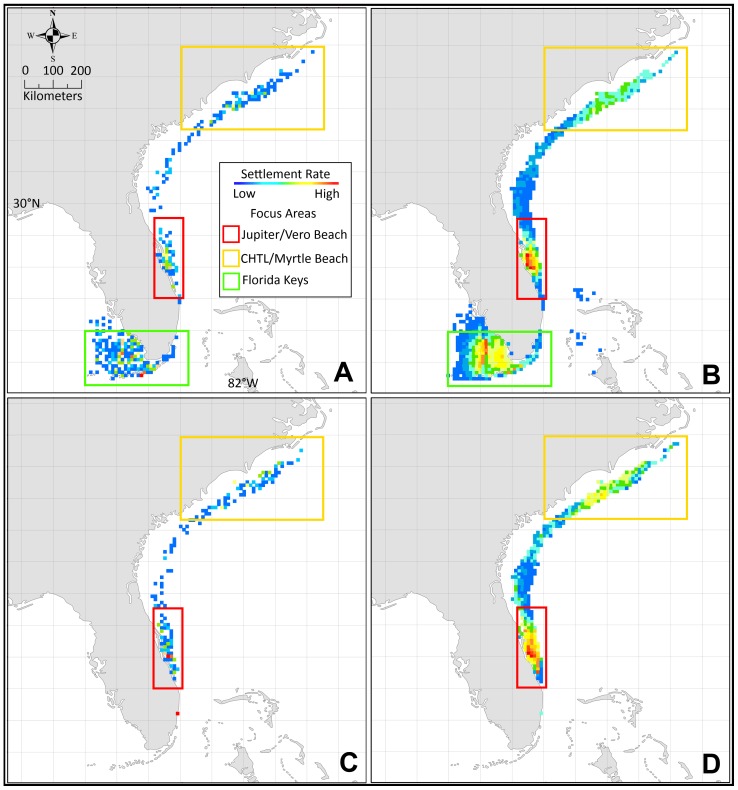
Sensitivity Analysis to Larval Mortality. Settlement rates of adult breeding populations for panther grouper on a ‘hot’ (red) to ‘cold’ (blue) scale using Jenks' natural breaks as class divisions. CS_FK_ with a larval mortality rate of 0.22 d ^−1^ (A), 0.18 d ^−1^ (B). CS_BC_ with a larval mortality rate of 0.22 d ^−1^ (C), 0.18 d ^−1^ (D).

When plotted on a map and summed by location, the results for each variation of *Z_p_* (±10%, 0.18 d ^−1^ and 0.22 d ^−1^) indicate the same general pattern of spread with the same ‘hot spots’ as observed in the original simulations (Figure 6). This implies that pattern and overall spatial distribution are not highly sensitive to *Z_p_*. The ρ values calculated using an alternate *Z_p_* of 0.18 d ^−1^ resulted in values of 0.63 (CS_FK_) and 0.86 (CS_BC_). Using a significance level of 0.05 and critical value of 0.59 (CS_FK_) and 0.65 (CS_BC_), ρ values for both alternate scenarios proved to be significantly correlated to the original RMs. These findings indicate that the actual pattern and sequence of spread is not greatly sensitive to *Z_p_* when the rate is decreased. Contrastingly, stark differences were noted in the count and concentrations of settled kernels, with a mean settled kernel count per simulation of 48 (CS_FK_) and 17 (CS_BC_) at a *Z_p_* of 0.18 d ^−1^ and 1071 (CS_FK_) and 506 (CS_BC_) at 0.22 d ^−1^. This indicates that quantity of settled kernels, a proxy for recruitment in the model, is highly sensitive to *Z_p_* and is in agreement with findings by [13]. Also noted was a decrease in mean settlement month per step for both case studies with an alternate *Z_p_* of 0.18 d ^−1^, which was especially prevalent in the last few steps of each invasion sequence. This result indicates that the projected invasions were gaining traction towards the end of the simulations. The alternate CS_FK,_ with a *Z_p_* of 0.18 d ^−1^, also displayed a potential crossover event to the Bahamas which was not projected in the original simulations, indicating a lower *Z_p_* could result in spread to the Bahamas at a faster pace (Figure 6B). Lastly, ρ values were not calculated for a *Z_p_* of 0.22 d ^−1^ as a result of greatly reduced numbers of settled kernels in both case studies. As a result, these scenarios were unable to reliably reproduce the step sequences displayed in the original case studies. This also demonstrates the sensitivity in the model to *Z_p_*.

## Discussion

### 4.1. Study Results

The ISM indicates several key locations which present a high likelihood for retention of larvae and the eventual development of breeding populations of panther grouper, given the constraints of the model. Common to all case studies, just south of the CHTL (a northernmost record of 34.817° N latitude was recorded in the model) near Myrtle Beach and north and west of Jupiter Florida centered close to Vero Beach, are high-risk areas. The Florida Keys/Ten Thousand Islands location is seen as lower risk with lower settlement rates. Based on the composite study, the highest likelihood of establishment of a breeding population of panther grouper, regardless of introduction point, is north of Jupiter Florida, centered near Vero Beach. Our modeling outputs suggest that Vero Beach is to be the highest priority for monitoring efforts, followed by the Myrtle Beach/CHTL and the Ten Thousand Islands area.

Based on the two individual case studies, a Florida Keys origin is most precocious as this would provide a conduit to the west coast of Florida and the Gulf of Mexico. If the invasion scenario follows the pattern documented by lionfish, this Florida Keys origin would short-circuit the progression stage sequence, allowing ingress of the panther grouper into the Gulf of Mexico much sooner than occurred with lionfish [4]. Based on life history and fecundity traits alone, it is likely that the lionfish may be more suited as an invasive species in the Atlantic than the panther grouper, however this does not preclude the possibility that the species will become established. The supposition that it may be a less robust invasion process than occurred with the lionfish is based on several key ecomorphological differences in panther grouper (including lack of venomous defenses, similarity to native groupers, and familiarity of predators and prey to the panther grouper body morphology) which are advantageous to the lionfish.

As anticipated, and in accordance with studies by [13], the model shows sensitivity to *Z_p_* regarding concentration and quantity of settled larvae in both case studies. This is consistent with literature indicating that recruitment in most fish population models display high sensitivity to larval mortality. Though this sensitivity affects settling rates and likely timing of an invasion in the ISM, the predicted pattern and location of high risk areas remain unchanged and are robust. Accordingly, the maps produced are useful as baseline guides for early detection efforts. Lastly, we anticipate that *Z_p_* above 0.22 d^−1^ will greatly decrease the chances of a successful invasion for the panther grouper, while lower *Z_p_* will likely increase the chance of successful establishment in the study area.

### 4.2. Potential Limitations of the ISM

Numerical models examining complex systems, such as the marine environment, suffer from uncertainty arising from the inevitable lack of a full understanding of the system. Approximation or underlying data errors or fundamental flaws in the model itself can introduce bias and undermine the model results. Acknowledging these limitations, this study aims to reduce inherent uncertainty within the model by eliminating bias when selecting origination locations and instead employs random locations within the study area. Additionally, the two case studies presented are validated against H_o_ and tested for precision using a standard metric, the SRCC. Sensitivity analysis is also performed to test model robustness to variances in larval mortality.

While the panther grouper has been found in the Florida Keys and Broward County, this does not confirm breeding populations. In both case studies, it is assumed that a breeding population persists at the origins and the lag period that is sometimes present with exotic invasions is ignored [24]. The model also overlooks occasional continued introductions which may contribute to the population and assumes neither infringement nor long-distance movement (greater than the model scale of 6 arc minutes) among sites of breeding populations. This species has shown site fidelity, decreasing the likelihood for site relocation as an influencing factor in the model results. The distribution of larvae is dependent upon passive movement of kernels within the model, and no adjustment is made for possible local retention of larvae at the origins. The ISM does not consider cyclical breeding cycles, though both peaks and year-round reproduction have been documented for panther grouper. While the model has been validated in the case of lionfish using a historical pattern, this invasion history is not present for the panther grouper. It is promising that the ISM algorithm has shown predictive capabilities in a previous study with a species of similar feeding ecology and breeding modality. Additional studies involving potential fecundity, mortality rates, and tolerances of panther grouper in the Atlantic would be helpful to adjust input values.

## Conclusion

This paper presents a rapid-response modelling study of the potential establishment and spread of the panther grouper in the western Atlantic in an effort to direct early detection and eradication efforts before the species has gained traction. This study identifies three areas of concern for potential establishment of the species in the western Atlantic, should a founder population occur in any location in the area examined (extending from approximately 29° N to 24° N on the Atlantic side of Florida in waters <40 m). These locations include; 1) just south of the Cape Hatteras Tropical Limit/Myrtle Beach, 2) north of Jupiter Florida/Vero Beach, and 3) the Florida Keys/Ten Thousand Islands location. As breeding populations are not yet thought to occur, it is suggested that these three locations should be high priority for monitoring and early detection efforts to prevent the proliferation of the panther grouper in the Atlantic. This study gives an early indication of potential hot spots of establishment to guide detection, containment, and perhaps eradication efforts.

## Acknowledgments

We thank the USGS for compiling panther grouper records and making them publically available as well as support from the National Coral Reef Institute (NCRI) at Nova Southeastern University for this study. We also thank Dr. Patricia Illoldi-Rangel and the additional anonymous reviewers whose guidance was invaluable. This is NCRI contribution 153.
