# Correction: Modeling the Potential Spread of the Recently Identified Non-Native Panther Grouper (*Chromileptes altivelis)* in the Atlantic Using a Cellular Automaton Approach

**DOI:** 10.1371/annotation/54b58291-1a3f-4a38-b48a-fe4466b7f61b

**Published:** 2013-09-19

**Authors:** Matthew W. Johnston, Sam J. Purkis

The current funding statement lists the NCRI contribution number as "XXX." The Funding statement should read, "This study was supported in part by the National Coral Reef Institute. This is NCRI contribution 153. The funders had no role in study design, data collection and analysis, decision to publish, or preparation of the manuscript." 

